# Methicillin-Resistant *Staphylococcus aureus* as a Probable Cause of Antibiotic-Associated Enterocolitis

**DOI:** 10.1155/2018/3106305

**Published:** 2018-08-05

**Authors:** Alison B. Lane, Nathanial K. Copeland, Fatma Onmus-Leone, James V. Lawler

**Affiliations:** ^1^Department of Medicine, Division of Infectious Diseases, Walter Reed National Military Medical Center, Bethesda, MD, USA; ^2^Multidrug-Resistant Organism Repository and Surveillance Network, Walter Reed Army Institute of Research, Silver Spring, MD, USA; ^3^Naval Medical Research Center, Frederick, MD, USA

## Abstract

Antibiotic-associated diarrhea is typically associated with *Clostridium difficile*. However, *Staphylococcus aureus* has also been described as a cause of antibiotic-associated enterocolitis and diarrhea and is likely an underrecognized etiology. We present a case of enterocolitis and urinary tract infection caused by methicillin-resistant *S. aureus* following antibiotic treatment.

## 1. Introduction

The most commonly identified pathogen in healthcare-associated diarrhea is *Clostridium difficile*, particularly in the setting of antibiotic use [1]. However, prior to the identification of *C. difficile* and its toxins as contributors to healthcare-associated diarrhea in the mid-1970s, *Staphylococcus aureus* was recognized as a causative agent of antibiotic-associated enterocolitis (AAE). Staphylococcal enterocolitis was first described in the 1950s, but increasing prevalence of *C. difficile* in recent years has led to underrecognition of *S. aureus* as an etiology of nosocomial and antibiotic-associated diarrhea [1]. We present a case of enterocolitis and urinary tract infection caused by methicillin-resistant *S. aureus* (MRSA) following antibiotic treatment in a MRSA-colonized patient.

## 2. Case

An 87-year-old woman presented to the emergency department with three days of abdominal pain, nausea, vomiting, and copious diarrhea. She described watery stools occurring up to eight times each day, without blood or mucus. She reported fatigue and anorexia but denied fevers and chills, as well as dysuria or other urinary symptoms. Six days previously, she had been discharged after a week-long hospitalization for ST-elevation myocardial infarction, which included diagnostic cardiac catheterization via femoral access. During this hospitalization, she was also diagnosed with community-acquired pneumonia and was started on intravenous ceftriaxone and azithromycin, with transition to oral levofloxacin at discharge.

On initial examination, she was afebrile with heart rate of 91 and blood pressure 95/48 (slightly below her outpatient baseline). Oral mucosae were dry and abdomen was soft, nondistended and diffusely tender without peritoneal signs, suprapubic tenderness, or costovertebral angle tenderness. Initial labs revealed a neutrophil-predominant leukocytosis of 15,800 cells/*µ*L; creatinine 1.31 mg/dL (previous baseline 0.65 mg/dL); and normal lactate, liver-associated enzymes, and lipase. Urinalysis demonstrated positive leukocyte esterase, 34 leukocytes/hpf, and negative nitrite. Fecal leukocytes and *C. difficile* PCR (Cepheid® Xpert® *C. difficile*) were negative. Abdominal plain films were unremarkable.

The patient was admitted for fluid resuscitation and symptomatic management with ondansetron and loperamide, with improvement of her abdominal pain and nausea. However, her profuse diarrhea persisted, and on hospital day 2, both urine and stool cultures obtained on admission grew MRSA. Abdominal CT revealed sigmoid bowel wall thickening consistent with colitis. The patient was started on vancomycin via both intravenous (750 mg daily) and oral (125 mg every 6 hours) routes with significant improvement of her diarrhea the following day. Though blood cultures remained negative, given her multifocal MRSA in the setting of recent femoral catheterization and history of rheumatic heart disease, she underwent transesophageal echocardiography which showed no evidence of endocarditis. She finished 10 days of oral and 14 days of parenteral vancomycin and recovered completely.

Due to the overwhelming predominance of MRSA in the stool culture and concomitant presence in the urine, isolates from both sources underwent genotyping with whole-genome sequencing. DNA was extracted and then sequenced using MiSeq Reagent Kit v3 (Illumina, San Diego, CA, USA). Sequencing reads were assembled, and comparative genomic analyses were performed using Geneious (Biomatters, Auckland, New Zealand) [2]. The isolates were found to be genetically identical and shared several genes potentially involved in pathogenesis (Table 1). Notably, the staphylococcal enterotoxin B (*seb*) gene and precursor gene for staphylococcal enterotoxin D (*entD*) were present.

## 3. Discussion

While MRSA is well-recognized as an important and serious nosocomial and community-acquired pathogen, its role as a causative agent of AAE is less established in recent Western literature. AAE caused by enterotoxin-producing staphylococci was initially described in the 1950s [3, 4]. However, in 1978, *Clostridium difficile* and its toxins were identified as the principle causative agents of post-antibiotic pseudomembranous colitis (*S. aureus* was also seen, though accounted for a minority of cases) [5, 6]. Since that time, authors have only rarely reported cases of MRSA AAE, with more cases published in Japanese [7] compared to Western literature [8]. However, a prospective study of stools negative for *C. difficile* toxin from patients with diarrhea found MRSA in 13 out of 3210 stool specimens, with 11 isolates producing staphylococcal enterotoxins [9]. Recently, Iwata et al. conducted a comprehensive review of the literature related to MRSA AAE and identified nine criteria that support a causative relationship [10]. It is therefore possible that MRSA constitutes an underappreciated cause of AAE [11].

Similar to *C. difficile* infection, risk factors for development of MRSA AAE include advanced age, immunosuppression, prolonged hospital stays, and previous antibiotic treatment [12, 13]. For MRSA in particular, prior fluoroquinolone use (as in our patient) is associated with an increased risk [12]. Though MRSA AAE can have a very similar clinical presentation to *C. difficile*, it is more likely to involve the small intestine instead of cecum or colon, and can result in localized bowel wall thickening on CT, as seen in this case [9, 13, 14]. Diarrhea is typically profuse, large volume, and watery, and patients with MRSA colitis are more likely to have associated symptoms of nausea, vomiting, and fever [13].

Similar to the pathogenesis of *C. difficile*, MRSA enterocolitis is likely caused by a toxin-mediated mechanism. More than 20 different staphylococcal enterotoxins (SEs) have been identified [15]. Many have well-understood roles in staphylococcal food poisoning, and several reports have identified TSST-1 as well as SE-A, B, C, D, and E in cases of *S. aureus* enterocolitis [7, 9, 10, 12]. Staphylococcal leucocidin LukE-LukD also has proposed involvement in the disease process via a cytotoxic mechanism, and in one case, series was identified in 94% of MRSA enterocolitis isolates [16]. The majority of MRSA are toxin-producing strains, which may account for their relative prevalence (in comparison with MSSA) in cases of enterocolitis [15].

In cases of severe antibiotic-associated diarrhea when *C. difficile* toxin testing is negative, stool culture should be performed for further evaluation, and isolation of MRSA as the predominant organism should suggest causation. Testing for viral enteric pathogens (though not performed in this case) is now more widely available, and these should also be excluded. When MRSA is identified or suspected as the cause of antibiotic-associated colitis, oral vancomycin is the recommended treatment [12]. While diarrheal symptoms often begin to resolve within 24 hours of initiation of vancomycin, typically a 10–14-day course of 125–250 mg daily is used to ensure adequate treatment, though data on exact dose and duration are sparse and warrant further study [12, 17]. Additionally, supportive care and symptomatic management are important, and as diarrhea tends to be very profuse, aggressive fluid resuscitation is often necessary.

Though *C. difficile* is the most common infectious cause of antibiotic-associated diarrhea, MRSA is a clinically relevant and likely underdiagnosed etiology. Our patient's presentation with colitis following a recent course of antibiotics, negative *C. difficile* PCR, MRSA overgrowth on stool cultures to the exclusion of normal fecal flora, and rapid resolution of diarrhea following initiation of oral vancomycin was consistent with MRSA as the inciting pathogen.

## Figures and Tables

**Table 1:** Putative and confirmed virulence and toxin genes and associated proteins detected in both MRSA isolates.

	Clinically relevant downstream effects
Toxin genes	
*entD*^a^, *seb*^a^	Staphylococcal enterotoxins (SE-B and D) which act as superantigens
*hla*, *hld*, *hlgA*, *hlgB*, *hlgC*	Cytotoxins
Virulence genes	
*aur*, *cap8A*, *cap8B*, *cap8C*, *cap8D*, *cap8F*, *cap8G*, *cap8L*, *cap8N*, *cap8M*, *cap8O*, *chp*, *geh*, *sbi*, *scn*, *sspB*, *sspC*	Disruption of host complement activity, phagocytosis, and/or immune cell chemotaxis
*ebpS*, *fnb*, *sdrC*	Host tissue adherence
*esaA*, *esaB*, *esaC*, *essB*, *essC*, *esxA*, *esxB*	Virulence proteins via unclear mechanisms, perhaps modulate host cell apoptosis
*hysA*, *sak*	Tissue degradation and invasion
*icaA*, *icaB*, *icaR*	Biofilm formation
*isdA*, *isdB*, *isdD*, *isdF*, *isdG*	Iron acquisition
MW0023	Immune modulation
*srtB*	Modification of bacterial surface proteins
*sspA*	Modification of bacterial enzymes and cleavage of immunoglobulin G

^a^Previously demonstrated in cases of MRSA antibiotic-associated enterocolitis.
